# Surface Plasmon Resonance as a Tool for Ligand Binding Investigation of Engineered GPR17 Receptor, a G Protein Coupled Receptor Involved in Myelination

**DOI:** 10.3389/fchem.2019.00910

**Published:** 2020-01-10

**Authors:** Davide Capelli, Chiara Parravicini, Giorgio Pochetti, Roberta Montanari, Caterina Temporini, Marco Rabuffetti, Maria Letizia Trincavelli, Simona Daniele, Marta Fumagalli, Simona Saporiti, Elisabetta Bonfanti, Maria P. Abbracchio, Ivano Eberini, Stefania Ceruti, Enrica Calleri, Stefano Capaldi

**Affiliations:** ^1^Istituto di Cristallografia, Consiglio Nazionale delle Ricerche, Rome, Italy; ^2^Department of Pharmacological and Biomolecular Sciences, Università degli Studi di Milano, Milan, Italy; ^3^Department of Drug Sciences, University of Pavia, Pavia, Italy; ^4^Department of Food, Environmental and Nutritional Sciences, Università degli Studi di Milano, Milan, Italy; ^5^Department of Pharmacy, University of Pisa, Pisa, Italy; ^6^Department of Pharmacological and Biomolecular Sciences, Data Science Research Center, Università degli Studi di Milano, Milan, Italy; ^7^Department of Biotechnology, University of Verona, Verona, Italy

**Keywords:** GPR17, G-protein coupled receptors, SPR, ligand binding, Cangrelor, Asinex 1

## Abstract

The aim of this study was to investigate the potential of surface plasmon resonance (SPR) spectroscopy for the measurement of real-time ligand-binding affinities and kinetic parameters for GPR17, a G protein-coupled receptor (GPCR) of major interest in medicinal chemistry as potential target in demyelinating diseases. The receptor was directly captured, in a single-step, from solubilized membrane extracts on the sensor chip through a covalently bound anti-6x-His-antibody and retained its ligand binding activity for over 24 h. Furthermore, our experimental setup made possible, after a mild regeneration step, to remove the bound receptor without damaging the antibody, and thus to reuse many times the same chip. Two engineered variants of GPR17, designed for crystallographic studies, were expressed in insect cells, extracted from crude membranes and analyzed for their binding with two high affinity ligands: the antagonist Cangrelor and the agonist Asinex 1. The calculated kinetic parameters and binding constants of ligands were in good agreement with those reported from activity assays and highlighted a possible functional role of the N-terminal residues of the receptor in ligand recognition and binding. Validation of SPR results was obtained by docking and molecular dynamics of GPR17-ligands interactions and by functional *in vitro* studies. The latter allowed us to confirm that Asinex 1 behaves as GPR17 receptor agonist, inhibits forskolin-stimulated adenylyl cyclase pathway and promotes oligodendrocyte precursor cell maturation and myelinating ability.

## Introduction

The molecular targets for about 50–60% of currently validated drugs are membrane proteins, such as G-protein coupled receptors (GPCRs); this class of proteins still features the main target in drug discovery programs (Hauser et al., 2017; Ribeiro-Oliveira et al., 2019). To this purpose, a range of chemical, biochemical and biophysical techniques are available for the characterization of ligand binding and for screening libraries of compounds searching for potential drug candidates. One of such techniques is surface plasmon resonance (SPR) spectroscopy, a label-free technique which enables measurement of real-time ligand-binding affinities and kinetics using relatively small amounts of membrane protein in a native or native-like environment (Olaru et al., 2015).

The typical SPR experimental protocol involves the direct binding of ligands on an immobilized target, which is usually a pure protein. Measuring the binding kinetics and affinities of ligands to intact membrane proteins by SPR is a challenging task, largely because of the inherent difficulties in capturing membrane proteins on chip surfaces while retaining their native conformation (Maynard et al., 2009; Patching, 2014) either in the original membrane environment or in membrane-mimicking structures, such as for example, lipid bilayer disks (Lundquist et al., 2010). An alternative method consists in capturing the detergent-solubilized receptor, engineered with a tag (such as multiple histidine residues or a short peptide sequence), with an appropriate antibody which has been previously covalently immobilized onto a chip through covalent bonding (Rich et al., 2011).

A gap still holds between the consolidated SPR technology and the request for innovative and robust immobilization methods for GPCRs. Indeed, in face of their importance as pharmacological targets, the intrinsic instability of GPCRs when extracted from the lipid milieu in the unligated form, makes them very challenging objects for SPR techniques, with only few examples of successful processing reported so far (i.e., neurotensin receptor type 1, chemokine receptor type 5 (CXCR5), β1 adrenergic receptor and purified adenosine 2A receptor) (Congreve et al., 2011; Adamson and Watts, 2014; Aristotelous et al., 2015; Bocquet et al., 2015; Chu et al., 2015).

Among the GPCR receptors, GPR17 holds a place of special interest in medicinal chemistry, being a key regulator of oligodendrocyte precursor cells (OPC) maturation (Fumagalli et al., 2016; Alavi et al., 2019). From a structural point of view, several binding sites have been described, which are the target of different classes of highly heterogeneous ligands (i.e., uracil nucleotides, cysteinyl leukotrienes, chemokines, and oxysterols) (Ciana et al., 2006; Eberini et al., 2011; Sensi et al., 2014; Parravicini et al., 2016). From a functional point of view, GPR17 is one of the most interesting targets for neurodegenerative disorders, since it crucially modulates the maturation of oligodendrocytes, the cells responsible for the production of myelin that, in turn, ensheates neuronal endings, thus allowing nerve impulse transmission. Myelin preservation and reconstruction accelerate neuronal repair and neurological recovery, and indeed represents a highly innovative approach to diseases, such as multiple sclerosis, Alzheimer's and cerebral ischemia (Fumagalli et al., 2015; Bonfanti et al., 2017; Seyedsadr and Ineichen, 2017). Therefore, new, more selective and pharmacokinetically suitable molecules are presently needed to translate promising preclinical data into patients care.

[^35^S]GTPγS binding-assay is an analytical method that has been used to investigate the activity of potential new GPR17 ligands with high accuracy and selectivity. The assay uses a GTP analog ([^35^S]GTPγS) which cannot be hydrolyzed by the GTPasic activity of the Gα subunit. Thus, GPCR activation or inhibition can be quantified by measuring the amount of radiolabelled GTP bound to the receptor-G protein complex (Harrison and Traynor, 2003). However, this standard functional assay does not provide any information on the direct molecular interaction between the ligand and the receptor.

In a previous work, frontal affinity chromatography-mass spectrometry (FAC-MS) was used to directly measure the direct binding of ligands to GPR17. In this assay, a liquid stationary phase containing membrane preparations from GPR17-expressing cells was coupled to an electrospray mass spectrometer as detector. Through the continuous infusion into the column of a solution of nucleotide analog it was possible to screen a high number of molecules in a single analysis, and to calculate the interaction between the receptor and different potential ligands (Calleri et al., 2010). Although this assay proved to be a powerful tool for the rapid screening of many compounds and for the assessment of low-to-medium affinity ligands, the approach is less effective in the characterization of the binding in the low nanomolar range.

Here, we report an SPR-based protocol that allowed us to efficiently immobilize two engineered variants of GPR17 on a sensor chip and to detect and measure the direct binding of two high affinity ligands. The calculated kinetic parameters and binding constants are in good agreement with those previously reported by activity assays and highlight a possible role of the N-terminal residues of receptor in ligand recognition.

## Materials and Methods

### Reagents and Instrumentation

Surface Plasmon Resonance (SPR) experiments with the ligands were performed at 25°C using a Pioneer AE optical biosensor (Molecular Devices-ForteBio) equipped with a PCH sensor chip (linear polycarboxylate hydrogel), and equilibrated with running buffer 20 mM Hepes, pH 7.5, 0.15 M NaCl, 0.03% Dodecyl Maltoside (DDM) and Cholesteryl Hemisuccinate (CHS) solution (DDM/CHS ratio 5:1) (with 1% DMSO, in the experiment with the agonist compound).

The GPR17 antagonist Cangrelor in its purified enantiomer (dichloro-[[[(2R,3S,4R,5R)-3,4-dihydroxy-5-[6-(2-methylsulfanylethylamino)-2-(3,3,3-trifluoropropylsulfanyl)purin-9-yl]oxolan-2-yl]methoxy-hydroxyphosphoryl]oxy-hydroxyphosphoryl]methyl]phosphonic acid) was a kind gift of Medicines Company (Parsippany, NJ, USA) and agonist Asinex 1 (2-[[5-(2-methoxyphenyl)-4-(4-methoxyphenyl)-4H-1,2,4-triazol-3-yl]thio]-N-phenyl-propanamide; CAS 483283-39-2, previously published as ASN 02563583), was purchased from Ambinter (c/o Greenpharma, Orlèans, France) as racemic mixture. The detergents were from Anatrace, the anti-His_6_ antibody and other laboratory reagents were from Sigma.

### Protein Engineering

The cDNA sequence of wild type human GPR17 receptor (short isoform, Uniprot id: Q13304-2) cloned into the pcDNA3.1 vector was used as template for DNA amplification. A modified cysteine-free version of T4 lysozyme (T4L) was inserted in the third intracellular loop (IC3), replacing residues 224–229, by short overlap extension (SOE) PCR. Briefly, three overlapping fragments encoding residues 1–223 of GPR17, 2–160 of T4L and 230–339 of GPR17, respectively were amplified by PCR and used as templates for a final round of amplification. The primers were designed to generate the full length (GPR17-T4 1-339) and a shorter variant lacking the first 15 amino acids (GPR17-T4 16-339) of the T4L chimeric receptor fused with a TEV cleavage site and a 10XHis-tag at the C-terminus. The two constructs were further modified by introducing the D293^7.49^N mutation using the QuikChange II Site-Directed Mutagenesis Kit (Stratagene). The resulting sequences were cloned into the pFastBac vector (Invitrogen).

### Expression and Purification of GPT17 Variants From Insect Cells

High titer recombinant baculovirus stocks (>10^8^ pfu/mL) of the two variants were obtained with the Bac-toBac TOPO Expression system (Invitrogen) in SF9 insect cells. For protein expression, suspension cultures of SF9 or High Five cells at density of 2 × 10^6^ cells/mL in suitable serum-free medium were infected with the virus at a multiplicity of infection (m.o.i.) of 3. After 48 h, cells were collected by centrifugation and resuspended in a hypotonic Lysis Buffer (LB) composed of 10 mM HEPES, pH 7.5, 10 mM MgCl_2_, 20 mM KCl added with a protease inhibitor cocktail (Roche). The cells were disrupted by Dounce homogenization in a glass potter, the membrane fraction collected by high speed (45,000 rpm) centrifugation for 45 min and washed twice with the same buffer. After an additional high salt wash (LB with 1 M NaCl), the membranes were resuspended in LB containing 20% glycerol at total protein concentration of 3 mg/mL, flash-frozen in liquid nitrogen and stored at −80°C until use. Purified membranes were thawed on ice in presence of 1 mg/mL iodoacetamide, 50 μM Cangrelor and protease inhibitors, mixed with an equivalent volume of 2× Solubilization Buffer (SB, 100 mM HEPES pH 7.5, 0.6 M NaCl, 2% DDM/0.4% CHS) and stirred at 4°C for 2 h. The insoluble material was removed by centrifugation and the supernatant, added with 20 mM imidazole, was incubated overnight at 4°C with TALON IMAC resin (Clontech) (0.5 mL of resin for 300 mL of initial cell culture). The resin was extensively washed with 15–20 column volumes of Wash Buffer (50 mM HEPES pH 7.5, 0.3 M NaCl, 0.05% DDM/0.01%CHS, 10 μM Cangrelor, 50 mM imidazole) and the bound proteins eluted with 2 column volumes of Elution Buffer (50 mM Hepes pH 7.5, 0.3 M NaCl, 0.05% DDM/0.01% CHS, 10 μM Cangrelor, 300 mM imidazole). The purity of the final preparations was checked by SDS-PAGE and Western Blot with an anti-His_6_ monoclonal antibody (Sigma). The apo proteins were purified with the same protocol in the absence of Cangrelor. The presence of glycosylation was assessed by PNGase F digestion on denatured samples (laboratory-made reagents). The monodispersity of GPR17 samples in absence or in presence of 10 μM Cangrelor was evaluated by size exclusion chromatography on a 10/30 G200 column equilibrated in 20 mM HEPES pH 7.5, 0.15 M NaCl, 0.05% DDM/0.01% CHS.

### Antibody Immobilization

An antibody specific for His_6_-tagged proteins was immobilized on the Chip surface using amine coupling chemistry (Jonsson and Malmqvist, 1992; Lundquist et al., 2010). Briefly, flow cells were activated for 4 min by injecting 40 μL of 1:1 ratio of 100 mM N-hydroxysuccinimide (NHS)/400 mM ethyl-3(3-dymethylamino) propyl carbodiimide (EDC). The antibody solution (50 μL of 100 μg/mL antibody and 300 μL of Na Acetate, pH 4.5) was then injected for 10 min at 10 μL/min, followed by a 70 μL injection of ethanolamine 1 M, pH 8.0, to block any remaining activated groups on the surface. Approximately 18,000 RUs of antibody were immobilized on the three channels of the sensor chip. The immobilization step was performed in HBS buffer (Hepes 20 mM, pH 7.4, NaCl 150 mM, Tween 20 0.005%).

### Capturing the Solubilized Receptor

Engineered 10xHis-tag GPR17 receptor, expressed from two different constructs (1–339 and 16–339) in two different cell lines (H5 and SF9), was captured on the antibody for the SPR experiments. The C-terminal 10xHis-tag, situated in the intracellular region of the receptor, can be captured by the antibody allowing the extracellular N-terminal part, involved in ligand recognition, to be free to interact with the ligand.

Briefly, a frozen aliquot of crude membrane extract (0.1 mL) was mixed with 5 μL of Protease Inhibitor Cocktail 100× (Sigma) and incubated on ice for 30 min. An equal volume of solubilization buffer 2× (100 mM Hepes, pH 7.5, 0.6 M NaCl, 2% DDM/CHS 10:2) was then added to the membrane extract and incubated 2.5 h at 4°C, under gentle rotation. The membrane preparation was then centrifuged for 30 min at 14,000 rpm and the supernatant collected. Crude cell supernatant (diluted 1/3 in MES 50 mM, pH 6.0, 1% DDM/CHS 5:1) was injected (flux 5 μL/min) across the PCH sensor (channels 1 and 3) where the anti-His_6_ antibody was previously immobilized. The chip surface was washed for several hours with running buffer (flux 150 μL/min) to remove non-specifically bound supernatant debris. Alternatively, few short injections (15 s, flux 50 μL/min) of NaCl 0.5 M in DDM/CHS 1% can be effective to quickly remove the debris. After the cleaning procedure, ~500 RU of solubilized receptor were captured on channels 1 and 3 (channel 2 was used as reference).

The stability of the protein-antibody surface was demonstrated by the flat baseline achieved at the beginning of each sensogram. Once immobilized on the chip surface, GPR17 was found to maintain its activity over at least 24 h. Afterwards, the receptor was easily removed by few injections of regeneration solution (50 mM NaOH injected for 30 s at 50 μL/min). After the regeneration step, the chip surface was ready for capturing freshly solubilized receptor for new experiments.

### Kinetic Analysis of Cangrelor and Asinex 1

The GPR17 antagonist Cangrelor and the agonist Asinex 1, dissolved in running buffer, were tested in serial dilutions for binding to GPR17, from 1 μM to 3.125 nM for Asinex 1 and from 2 μM to 7.81 nM for Cangrelor (see Supplementary Figures S1–S4). Analytes were injected at a flow rate of 25 μL/min for 1 min over the three channels. Several buffer blanks were injected for double referencing. The regeneration of the surfaces between binding cycles was not necessary because the analytes completely dissociate in the 240 s dissociation phase. A DMSO calibration was performed for Asinex 1 [0.5–1.5% (vol/vol) DMSO] to correct for bulk refractive index shifts (Frostell-Karlsson et al., 2000). All the experiments were carried out in duplicate. All sensorgrams were processed by using double referencing (Myszka and Morton, 1998). Formation of the complex between GPR17 and ligands was indicated by the increase in resonance units (RUs) relative to baseline upon injection of each compound at each concentration (Supplementary Figures S1–S4). To obtain the kinetic and affinity constants the corrected response data were fed to the program QDAT. A kinetic analysis of each ligand/analyte interaction was obtained by fitting the response data to a 1:1 bimolecular interaction mode (Figures 3A–D). Constants reported in the table of Figure 3 represent the average of two independent analyses of each GPR17/analyte interaction.

### *In silico* Molecular Modeling and Ligand Docking

All the computational procedures, except for the molecular dynamics (MD) simulations, were carried out with the Molecular Operating Environment software (MOE2019.0101 Chemical Computing Group, Montreal, Canada), using the Amber12:EHT force field with the reaction field electrostatics treatment. The MD simulation of the GPR17-T4 1-339 variant and the procedures required for the preparation of the system were performed using the Schrödinger suite (Schrödinger, New York, NY, 2018).

#### Homology Modeling

The homology modeling procedure was performed using the MOE “Homology Model” program, starting from a multiple sequence alignment of the primary structures of a subgroup of structurally related class-A GPCRs, as previously described (Sensi et al., 2014; Parravicini et al., 2016). The multiple sequence alignment was performed using the TM-Coffee algorithm, a module of the T-Coffee package optimized for transmembrane proteins (Chang et al., 2012).

The tridimensional structure (3D) of the human GPR17 receptor in its wild-type form was built by comparative modeling, using as template the 2.7 Å resolution X-ray structure of the human P2Y1 receptor deposited in RCSB Protein Data Bank [PDB, code: 4XNW (Zhang et al., 2015)]. The GPR17-T4 1-339 variant was generated by a chimeric approach according to the above alignment, based on its engineered primary structure, using the structure of P2Y1 for modeling residues from Thr19-Leu223 and Lys230-Gal290, the structure of the C-X-C chemokine receptor type 4 (CXCR4) construct for modeling the T4 lysozyme (T4L) fusion, and the structure of the apelin receptor for modeling C-terminal domain (residues from Ala29 to Lys315) after structural alignment of the templates. The specific setting “C-terminal and N-terminal outgap modeling” was selected to model the N- and C-terminal regions from the full-length GPR17 sequence.

#### Low Mode Molecular Dynamics Search

N-terminal conformational search was performed using the MOE “LowMode MD” search method, by associating different conformational freedom to different regions of the protein, to speed-up calculations. In detail, residues from 1 to 19, 20 to 24, 25 to 40/80 to 115/157 to 202/248 to 286 (the upper TM bundle) and 41–79/116–156/203–247/287–319 (the upper TM bundle), were treated as a rigid body, flexible, fixed and inert. Also, the T4L was treated as inert. The Low Mode MD was carried out with standard settings, except for strain energy cutoff, which was set at 100 kcal/mol.

#### Ligand Docking

Molecular docking simulations were carried out using the MOE “Dock” program of the “Simulation” module, with a multi-step procedure useful for a more accurate estimation of the ligand binding free energy, as previously described (Eberini et al., 2011; Platonova et al., 2017). The GPR17 binding site was identified through the MOE “Site Finder” module. The receptor was treated as rigid for the docking calculations, while conformational space was sampled for ligands. Briefly, for each ligand 20,000 conformations were generated by sampling their rotatable bonds and placed using the Triangle Matcher methodology. Duplicate complexes were removed, and the accepted poses (1,000 for each ligand), were scored according to the London dG empirical scoring function, for an estimation of their binding free energy (Naïm et al., 2007). The 100 top scoring complexes for each ligand were submitted to a more in-depth refinement step based on molecular mechanics (MM), in which the final binding free energy was evaluated using the force-field based GBVI/WSA ΔG empirical scoring function to account for solvation effect (Wojciechowski and Lesyng, 2004). Only the 10 top-scoring complexes were kept at this stage. The “LigX” procedure was finally applied to the top-scoring pose to minimize *via* molecular mechanics (MM) both the ligand and the receptor binding site for a more accurate estimation of ligand affinity. Dissociation constant (Kd) values computed through this method have accuracy in the range of one order of magnitude (Eberini et al., 2008; Galli et al., 2014).

#### Molecular Dynamics

The preparation of the GPR17-T4 1-339 variant and its MD simulation were performed using the Desmond software, implemented in the Schrödinger suite, version 2019.2 (Desmond Molecular Dynamics System of the D. E. Shaw Research, New York, NY). Downstream residues belonging to the C-terminal portion of the receptor (Gly320-Leu339) were removed for MD simulations. The GPR17-T4 1-339 model was processed with the “Protein Preparation Wizard” tool in order to assign protonation states at pH of 7.0, cap C-terminal, optimize H-bond assignment, and minimize the protein energy. Then, the model was embedded in an explicit membrane bilayer using the “System Builder” tool. In detail, the receptor was oriented into membrane according to the OPM server prediction (Lomize et al., 2012) into a membrane model of 123 1-palmitoyl-2-oleoyl-sn-glycero-3-phosphocholine (POPC) and solvated with 11,104 TIP3P water molecules, in a cubic box with dimensions of 82 × 71 × 110 Å. The system was neutralized by adding 21 chloride ions and sodium chloride was added up to 0.1 M concentration. Prior to simulation, the system was energy-minimized and equilibrated through the relaxation protocol. The OPLS3 forcefield was used for both the MD relaxation phase and the productive MD simulations. For the membrane relaxation phase, the default protocol was applied; the productive MD was carried out for a total simulation time of 500 ns, with a recording interval for each frame of 500 ps resulting in a total of 1,000 recorded frames. The MD was performed under the following conditions: 300 K and 1.01325 bar with NPT ensemble class, Langevin as thermostat and barostat method with relaxation time of 1 and 2 ps, respectively, semi-isotropic pressure coupling style, RESPA integrator with timestep for bond-, short- and long- range bond interactions of 2, 2 and 6 fs, respectively, and 9 Å as cutoff for short range Coulombic interactions.

Analyses of the trajectories were performed with both Visual Molecular Dynamics (VMD) analysis tool (Humphrey et al., 1996) and GROMACS through VMD plugins (Berendsen et al., 1995).

### *In vitro* Assays on Cell Cultures

The Università degli Studi di Milano–La Statale (Italy) is compliant with all applicable national (D.Lgs. 26/2014) and European (Directive 2010/63/EU) regulations, for using animals in scientific research. All the experiments were approved by the Animal Care Committee of the Università degli Studi di Milano–La Statale, which is legally entitled for the use of animals for scientific purposes and by the Italian Ministry of Health (Authorization #473/2015-PR, 05/06/2015).

The pharmacological profile of Asinex 1 was assessed in three different *in vitro* assays of increasing complexity in oligodendrocyte precursor cells (OPCs) natively expressing GPR17. Primary rat OPCs were cultured either alone or in the presence of mouse DRG neurons, according to a well-established protocol, previously described in Fumagalli et al. (2015). The endogenous GPR17 ligands UDP and LTD_4_ were selected as reference ligands. For more details on culture preparation and analysis, see Supplementary Information.

#### cAMP Assay

In order to determine the intracellular cAMP levels, primary purified OPCs were treated with Asinex 1 after 6 days in culture, when GPR17 expression reaches the maximum peak. A competitive protein binding method was used following the procedure previously described (Fumagalli et al., 2015). Briefly, culture medium was removed, and cells were incubated at 37°C for 15 min with 400 μL of Neurobasal medium in the presence of the phosphodiesterase inhibitor Ro20-1724 (20 μM). The concentration-response curve of tested ligands was assessed by measuring their ability to inhibit cAMP accumulation stimulated by 10 μM forskolin. Asinex 1 was added to cells for 15 min at graded concentration (0.1–50 nM). When required, cells were preincubated for 10 min with the antagonist Cangrelor. Reactions were terminated by medium removal and addition of 200 μL of 0.4 N HCl. After 30 min, lysates were neutralized with 50 μL of 4 N KOH, and the suspension was centrifuged at 800× g for 5 min. For determination of cAMP, cAMP-binding protein isolated from bovine adrenal glands was incubated with [^3^H]cAMP (2 nM), 50 μL of cell lysate or cAMP standard (0–16 pmol) at 4°C for 150 min, in a total volume of 300 μL. Bound radioactivity was separated by rapid filtration through GF/C glass fiber filters and washed twice with 4 mL of 50 mM Tris-HCl, pH 7.4. Radioactivity was measured by liquid scintillation spectrometry.

#### Differentiation and Myelination Assays

GPR17 ligands were tested both in primary OPCs culture and in OPC/DRG co-cultures to assess their pro-differentiation and pro-myelination effect, respectively. According to the type of culture, a specific protocol of treatment was set up. For primary OPCs, cells were seeded on 13 mm poly-D,L-ornithine-coated coverslips (2 × 10^4^ cells/well) and maintained for 2 days in Neurobasal medium supplemented with B27 and proliferative factors (PDGF-BB and bFGF). Afterwards, OPC differentiation was induced by removing proliferative factors and by adding T3 and pharmacological treatments with Asinex 1 (1 or 10 nM) and LTD_4_ (200 nM), selected as reference compound, were performed the following day. After 48 h, cells were fixed for immunocytochemistry (ICC). For OPC/DRG co-cultures, Asinex 1 (10 nM) and UDP (100 μM) were added to cultures at day 4. The pharmacological treatment was repeated every 2 days up to day 15, when cells were fixed for ICC analysis. For more details, see Supplementary Information.

## Results

### Expression and Characterization of Engineered GPR17 Variants

In order to identify one or more GPR17 variants suitable for structural studies, we prepared and tested different engineered constructs of the receptor. A major bottleneck in structure determination of GPCRs by X-ray crystallography is obtaining of large amounts (>1–2 mg) of highly pure, homogeneous protein samples that are stable in detergent solutions when extracted from the lipid environment of the membranes. In the last decade, a number of protein engineering approaches have been used to overcome the problem of the intrinsic instability and conformational heterogeneity of GPCRs. These include truncations, site-directed mutagenesis, stabilization of the receptor with specific protein-binding partners (FABs and nanobodies) and the creation of chimeric constructs in which flexible loops are replaced with small soluble proteins that can favor the crystallogenesis process (Piscitelli et al., 2015). Inserting T4 lysozyme (T4L) in the third intracellular loop (ICL3) allowed to obtain the first high resolution crystal structure of β2-adrenergic receptor (Cherezov et al., 2007; Rosenbaum et al., 2007); the same strategy was subsequently used for other types of class A GPCRs (Wu et al., 2010; Granier et al., 2012; Zhang et al., 2012). More recently, the use of other fusion partners, such as thermostabilized BRIL or rubredoxin have proven effective in obtaining diffraction quality crystals of purinergic receptor P2Y_12_ (Zhang et al., 2014) and P2Y_1_ (Zhang et al., 2015).

**Figure 1:**
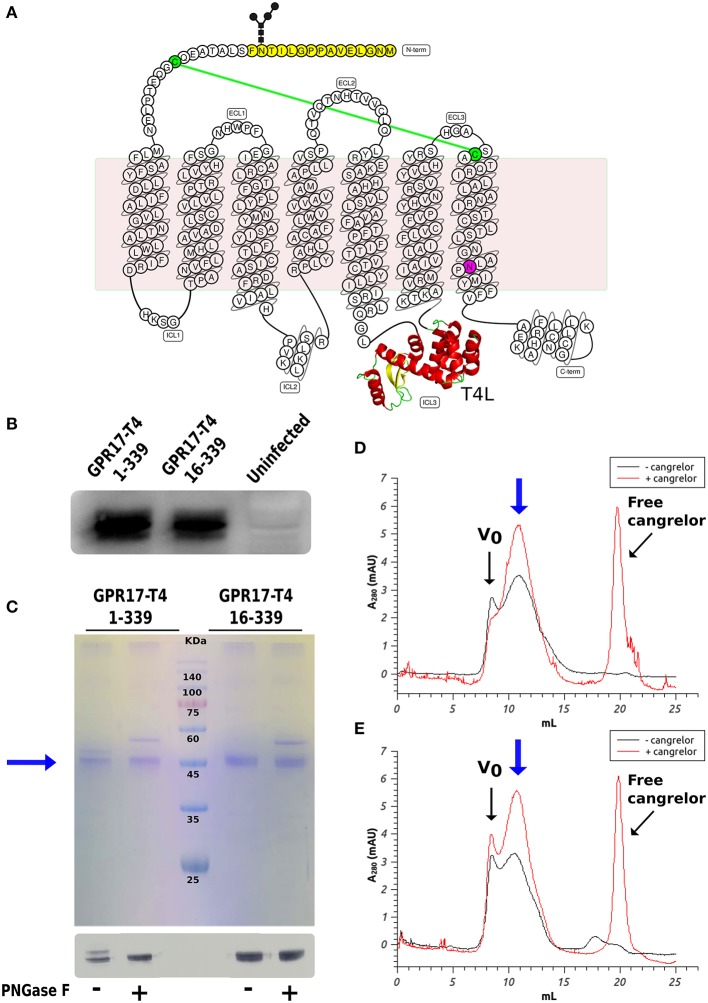
Expression and characterization of GPR17 engineered variants. **(A)** Snake-plot of modified GPR17. The first 15 amino acids (with N-glycosylation on Asn14) removed in the shorter construct are highlighted in yellow and the D293N mutation in purple. The T4 lysozyme inserted in ICL3 is represented in ribbon model. The two cysteines linked by disulfide bridge are colored in green. **(B)** Western blot analysis of GPR17 variants expression in High Five cells. Same amounts of whole cell extracts were probed with anti-His_6_-HRP conjugated antibody. **(C)** SDS-PAGE (top) and western blot (bottom) of purified GPR17-T4 1-339 and GPR17-T4 16-339 before and after treatment with PNGase F. **(D,E)** SEC profiles of purified GPR17-T4 1-339 **(D)** and GPR17-T4 16-339 **(E)** in absence or in presence of a saturating concentration of Cangrelor. The void volume is indicated with Vo.

We initially prepared a number of modified variants of GPR17. The modifications included a combination of truncations in different positions near the N- and C- termini and the fusion of the T4L moiety either at the N-terminal or in ICL3. All these constructs were used to generate recombinant baculoviruses for the expression of the receptor in insect cells. Insect cells are the most common expression system for eukaryotic membrane proteins and have been used for the production of most of the mammalian GPCRs crystallized so far (Schneider and Seifert, 2010; Milić and Veprintsev, 2015). Small scale expression trials identified two constructs that could be produced with a good yield in SF9 cells: the full-length receptor with T4L inserted in ICL3 (GPR17-T4 1-339) and a shorter version lacking the first 15 amino acids (GPR17-T4 16-339). The expression levels were further increased by mutating the conserved Asp^7.49^ (D293) into Asn, as described for other homologous P2Y receptors (Zhang et al., 2014, 2015). The modifications introduced in these two engineered variants are highlighted in the snake-plot displayed in Figure 1A. After optimization of the expression conditions, the two variants reached a maximum expression level of about 1–2 mg of receptor per liter of culture in both SF9 and High Five cells, with a slightly higher yield in the latter cell line (Figure 1B).

The two variants can be effectively extracted from the isolated membranes with a detergent mix composed of DDM and CHS and can then be purified by immobilized metal affinity chromatography (IMAC) exploiting the C-terminal His-tag as described in the Material and Methods (Figure 1C). A potential N-glycosylation site (Asn14) is present in the full-length construct and in fact the protein migrates as a double band in SDS-PAGE. The higher band disappeared upon treatment with PNGase F, indicating that a fraction of the receptor is actually glycosylated. Conversely, the shorter version 16–339 displays only a single well-defined band.

It is well-known that the binding of a high affinity agonist or antagonist stabilizes the GPCR in a specific signaling conformation, and increases the stability and enhances the size monodispersity of the receptor in detergent solution (Bertheleme et al., 2013; Grisshammer, 2017). We therefore assessed the effect of the antagonist Cangrelor on the two purified variants by size exclusion chromatography (SEC). Figures 1D,E summarize the results for GPR17-T4 1-339 and GPR17-T4 16-339, respectively. In both cases, the presence of Cangrelor increased the fraction of monodisperse receptor (the peak indicated by the blue arrow in the panels) and reduced the amount of aggregates that elute in the void volume, suggesting that these two variants retain the ability to bind the antagonist with comparable affinity. Although the use of a stabilizing ligand will be necessary to maximize the yield in large scale purifications, the presence, albeit in smaller quantities, of monodisperse apo protein in detergent solution makes these two constructs suitable for binding experiments and fragment screening by SPR.

### Molecular Modeling and Ligand Docking

In line with the increasing availability of class-A GPCR templates, thanks to homology approaches on GPR17, our previous studies have characterized the interactions of GPR17 with its known endogenous ligands (Parravicini et al., 2008, 2010, 2016; Sensi et al., 2014). We also identified a first set of entirely new and highly diverse GPR17 ligands, including the Asinex 1 compound which was identified within the Asinex Platinum Collection database (Calleri et al., 2010; Eberini et al., 2011).

**Figure 2:**
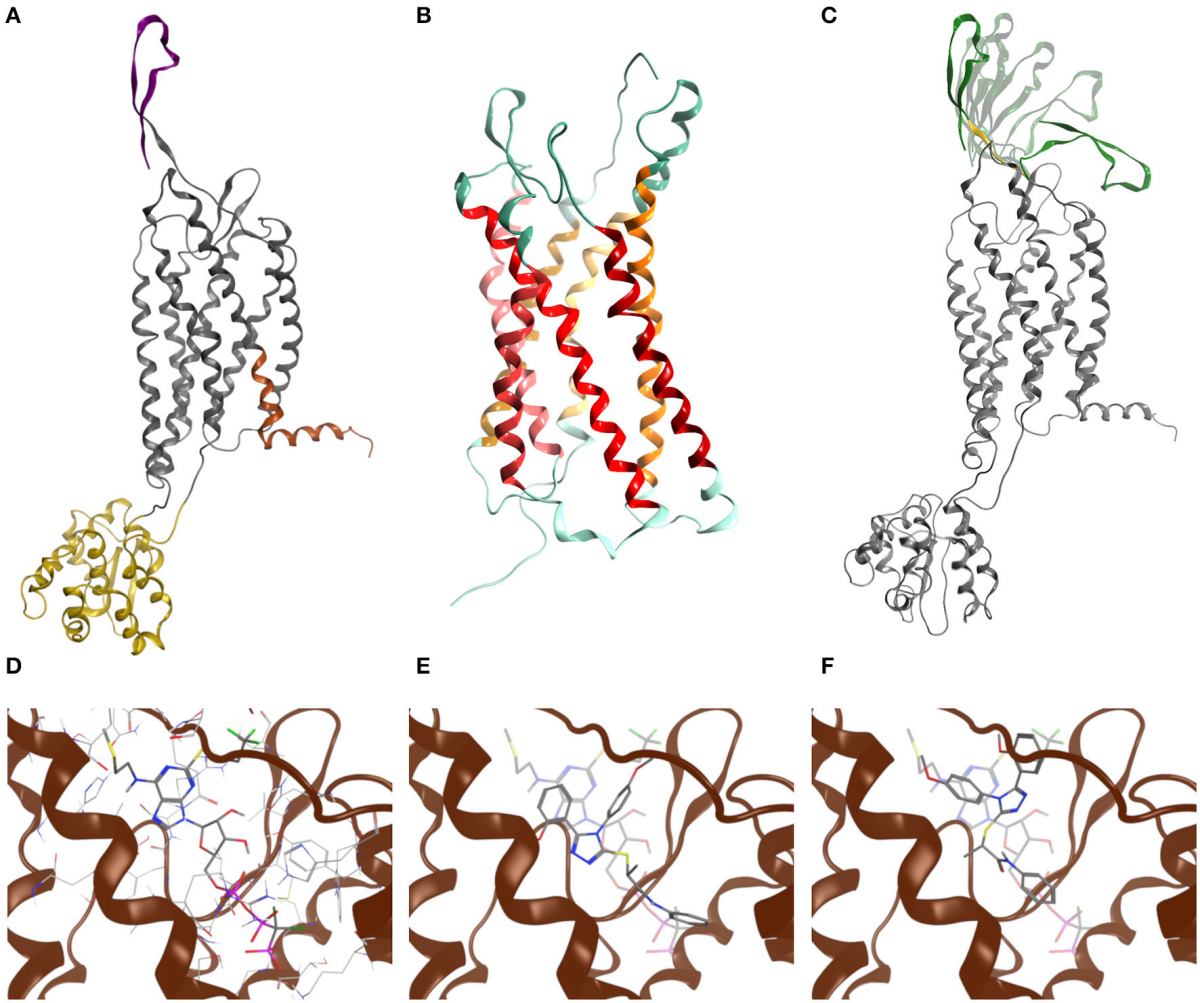
Molecular modeling of GPR17. **(A)** Chimeric model of GPR17-T4 1–339 (D293N) variant. Topological domains modeled with reference to different templates, namely P2Y_1_, CXCR4, and APJ receptor, are represented as gray, yellow, orange ribbons, respectively. The N-terminal region is purple. **(B)** Homology model of GPR17 based on P2Y_1_ receptor as template. Ribbons are colored according to MOE GPCR annotation. **(C)** Most representative N-terminal conformations after Low Mode MD sampling. During the Low Mode MD, green ribbons are treated as rigid bodies, yellow ribbons as free to move and gray ribbons as fixed or inert. **(D)** Cangrelor binding mode. **(E)** Asinex 1 binding mode (R enantiomer). **(F)** Asinex 1 binding mode (S enantiomer). In **(E,F)**, Asinex 1 and Cangrelor docking poses are superposed to hint to the basis of their competitive antagonism.

To evaluate whether the engineered GPR17 constructs are thermodynamically stable and still able to recognize ligands, a chimeric model of the full length GPR17-T4 1-339 variant was generated by replacing, during the homology modeling procedure, the ICL3 with the coordinates of the T4 lysozyme (T4L) fusion of CXCR4 structure (Wu et al., 2010). The structure of this construct is shown in Figure 2A. For validating the homology modeling procedure, besides the engineered GPR17 variant, an entirely new GPR17 model was built (Figure 2B), based on the recently solved X-ray structure of the P2Y_1_ receptor (Zhang et al., 2015). In this model, only the three-dimensional structure (3D) of residues between Thr19 and Leu311 was predicted, whereas the highly flexible N and C termini were not generated, since they are not included in the template. This GPR17 model resulted much more accurate than the previous ones, due to the closest sequence identity of GPR17 with respect to P2Y_1_, compared to that shared with all the other available crystallographic templates among related class-A GPCRs (26.4%). A pairwise alignment identity matrix between GPR17 and the primary structures of other related class-A GPCR, whose structure has been solved (Gacasan et al., 2017), is reported in Supplementary Figure S5.

Structural alignment of the helical bundle of the two homology models resulted in a low value of root-mean-square deviation (RMSD), corresponding to 1.4 Å (Supplementary Figure S6), suggesting that the use of the T4L as fusion partner does not impair the general architecture of the receptor as also found for other crystallized GPCRs (Wu et al., 2010).

As loop modeling for such flexible regions is known to be rather inaccurate, putative conformations of the N-terminal region were computed through an efficient conformational sampling approach based on the Low Mode MD. Low Mode MD generated only 20 conformations out of the 10,000 allowed for the N-terminal region, some representative of either the “open” or the “closed” state of the receptor (Figure 2C), confirming the ability to efficiently explore the conformational space of the test region. As shown in Table 1, all the 20 conformations are associated with negative values of potential energy (E), ranging from −299.53 to −205.17 kcal/mol; values of ΔE between the lowest- and the highest-energy conformations lower than 100 kcal/mol suggest that all conformations are stable and biologically relevant.

**Table 1:** Low mode MD conformations of the GPR17-T4 1-339 variant.

***E* (kcal/mol)**	**Δ*E* (kcal/mol)**	**Gyration radius (Å)**
−220.96	78.58	31.28
−225.73	73.80	31.29
−222.81	76.72	31.44
−208.99	90.55	31.56
−246.92	52.61	31.58
−217.04	82.50	31.62
−205.17	94.36	31.66
−211.98	87.56	31.68
−218.97	80.56	31.71
−235.83	63.70	31.78
−238.30	61.24	31.85
−212.94	86.59	31.87
−248.32	51.21	31.92
−258.03	41.50	32.05
−220.62	78.91	32.11
−252.47	47.06	32.12
−219.77	79.76	32.19
−282.52	17.02	32.25
−228.87	70.67	32.28
−299.53	0.00	32.33

The Low Mode MD conformation associated with the highest (32.3 Å) and lowest (31.3 Å) gyration radius were selected for further investigation as representative of the N-terminal open and closed form, respectively (Table 1).

The closed conformation was then submitted to a classic MD simulation performed in a heterogeneous water/membrane native-like environment, allowing unrestrained conformational sampling for the whole protein and assessing its stability over the simulation time. Overall, the analysis of the evolution of both geometrical and energetic parameters over 500 ns of MD simulation suggests that the GPR17-T4 1–339 variant is thermodynamically stable and confirms that the presence of intracellular T4L does not affect extracellular architecture of the receptor. However, as expected due its intrinsic flexibility, the N-terminal region is subject to large conformational changes during MD simulations (Supplementary Figure S7).

To assess the ability of the GPR17 construct to recognize ligands, the two synthetic compounds Asinex 1 and Cangrelor were docked into the binding site of both GPR17 “open” and “closed” form. For Asinex 1, both the R and S enantiomers were analyzed, since no information on the enantiomeric ratio is available from the vendor.

In line with previously published data (Eberini et al., 2011), both ligands were able to bind to the orthosteric binding site of GPR17 with high affinity (Table 2), supporting the evidence of a competition between the agonist Asinex 1 and the antagonist Cangrelor for the same site. Differences in binding free energy values were found between the docking complexes obtained with the open and closed conformations of GPR17. Indeed, all the ligands showed an increased affinity for the closed receptor conformation (Table 2). To account for solvent contribution, the Generalized Born implicit solvent model was applied for computing accurate ligand affinity after relaxation of each complex binding site. Since both ligands are characterized by highly hydrophobic moieties, it can be hypothesized that, in the open conformation, the exposure of these groups to the extracellular aqueous solvent negatively contributes to the binding free energy of the complexes, resulting in a loss in affinity in comparison with the closed form. A 2D plot depicting all the interactions engaged by the ligands as well as the ligand/receptor groups exposed to solvent is shown in Supplementary Figure S8. Figures 2D–F report superposition of the top-scoring poses obtained for Cangrelor and Asinex 1, respectively.

**Table 2:** Binding free energy values computed through molecular docking for GPR17-T41-339.

	**Docking score (kcal/mol)**	**LigX** ** (kcal/mol)**	**Kd [M]**	**pKd**
**OPEN CONFORMATION**				
Cangrelor	−10.23	−13.84	6.5295E-11	10.19
Asinex 1 (R)	−8.81	−9.64	8.049E-08	7.09
Asinex 1 (S)	−8.71	−9.24	1.5853E-07	6.80
**CLOSED CONFORMATION**				
Cangrelor	−10.76	−14.50	2.1339E-11	10.67
Asinex 1 (R)	−9.06	−10.07	3.8842E-08	7.41
Asinex 1 (S)	−8.91	−9.44	1.1296E-07	6.95

### SPR Experiments

**Figure 3:**
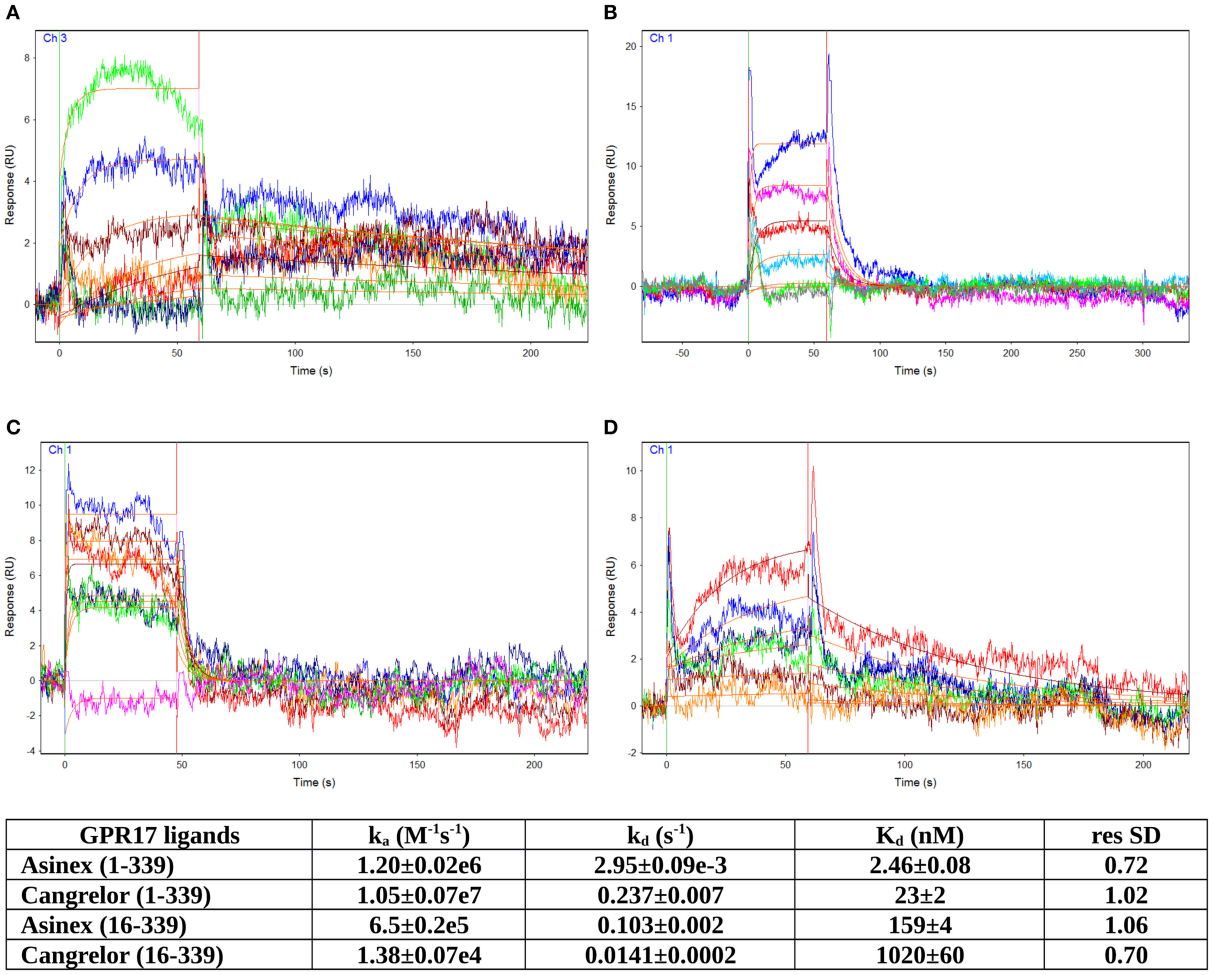
SPR analyses of ligand binding. Full kinetic analysis of the agonist Asinex 1 **(A,B)** and the antagonist Cangrelor **(C,D)** binding to GPR17-T4 1–339 and GPR17-T4 16–339, with the determined binding parameters listed in the table.

GPCRs are typically highly unstable when extracted from the cell membranes, making them particularly difficult to study for ligand screening with biophysical methods. Here, we used an effective protocol which allowed us to extract from crude membranes two engineered variants of GPR17 that are relatively stable in the ligand-free state, and to immobilize them on the surface of a sensor chip for SPR analysis. The SPR results obtained with the high affinity antagonist Cangrelor and the agonist Asinex 1 demonstrated that the immobilized GPR17-T4 variants retained their ability to specifically bind the analytes. Full kinetic analysis of the agonist Asinex 1 and the antagonist Cangrelor, when binding to GPR17-T4 (constructs 1–339 and 16–339) is shown in Figure 3, with calculated binding parameters listed in the table.

Although the ratio between Cangrelor and Asinex 1 affinities measured by SPR is similar to that reported in the literature (Abbracchio et al., 2006; Ciana et al., 2006; Eberini et al., 2011) (9.35 vs. 7), the absolute Kd values are 20–30 times higher in the SPR experiments. This discrepancy might be ascribed to some residual non-specific binding to GPR17-T4, due to the injection of crude membrane preparation, which could affect the calculated Kd values. In addition, a perfect correspondence should not be expected because SPR technique provides direct binding data, whereas the literature data refer to a functional assay that evaluates the ability of the agonist (Asinex 1) to increase the binding of [^35^S]GTPγS to the activated receptor or the ability of the antagonist (Cangrelor) to counteract the effect induced by either Asinex 1 (Eberini et al., 2011) and a reference agonist (Ciana et al., 2006).

Interestingly, the affinity of both ligands toward the full-length GPR17-T4 1–339 is higher (ca. 50 times) with respect to that of the N-terminal truncated form (construct 16–339), suggesting a possible role of the N-terminal region in the binding with the receptor.

### *In vitro* Assays on Primary OPCs

#### Asinex 1 Inhibits Forskolin-Stimulated Adenylyl Cyclase Activity in Primary OPCs

**Figure 4:**
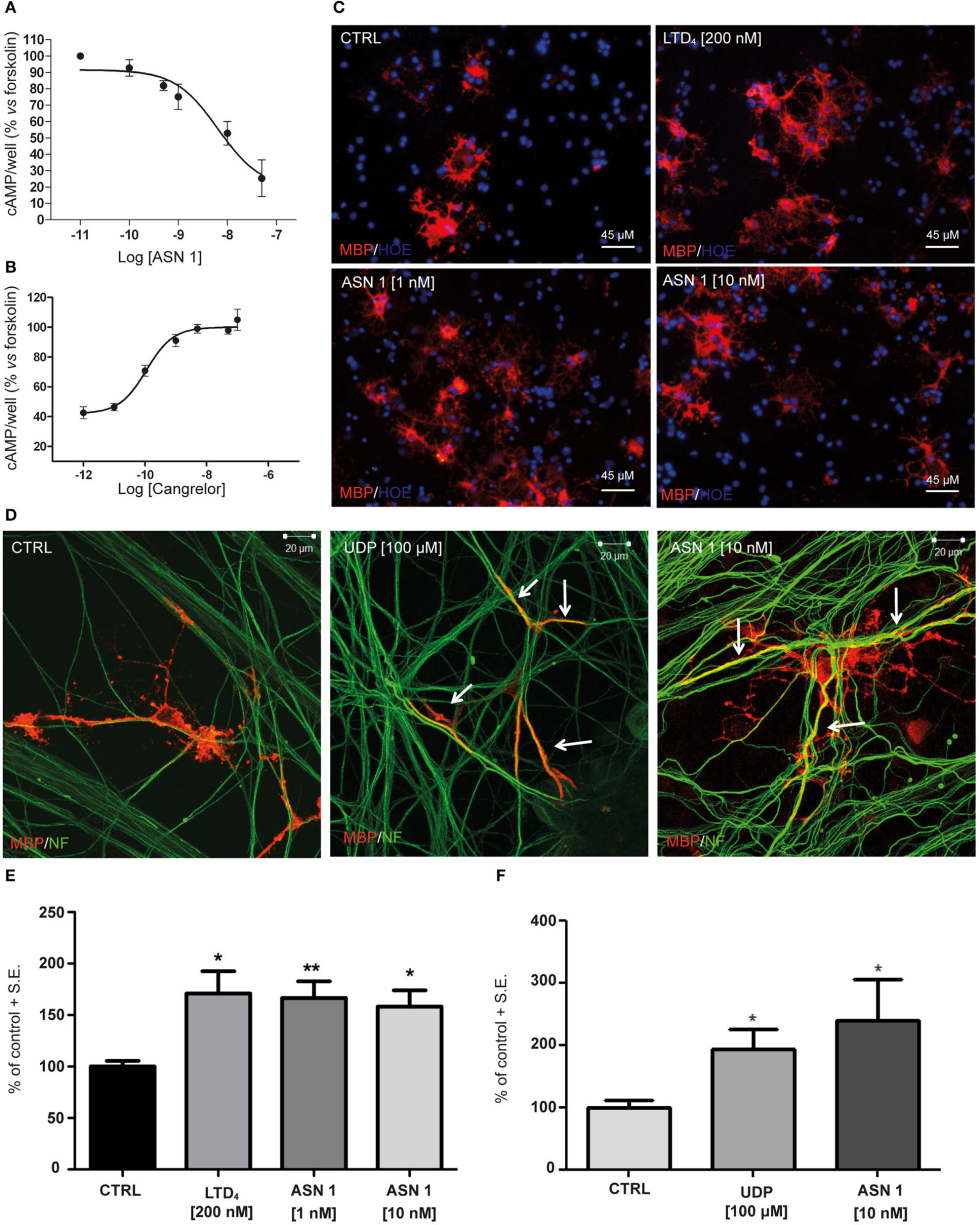
Functional validation of and SPR data on primary OPCs. **(A)** Inhibition of forskolin-stimulated adenylyl cyclase activity in primary OPCs by graded concentrations (0.1–50 nM) of the GPR17 agonist Asinex 1 (ASN 1). **(B)** The GPR17 antagonist Cangrelor concentration-dependently counteracts ASN 1-mediated inhibition of forskolin-stimulated adenylyl cyclase activity in primary OPCs. Cangrelor was used in the presence of a constant ASN 1 concentrations of 10 nM. Results are expressed with reference to forskolin-stimulated cAMP levels, set to 100%. Data represent the mean ± S.E. (error bars) of three separate experiments, each performed in duplicate. **(C)** Representative images of CTRL, LTD_4_-, ASN 1- (1 and 10 nM) treated OPCs, showing cell labeling with anti-MBP antibody (in red). Nuclei were labeled with Hoechst 33258 dye (HOE, in blue). Scale bars: 45 μm. **(D)** Representative images of control (CTRL), UDP- and ASN1-treated OPC-DRG co-cultures showing double immunostaining for anti-MBP antibody (red) and anti-neurofilament antibody (NF, green). Yellow color shows co-staining. Scale bars: 20 μm. **(E)** Histograms show quantification of the percentage of MBP+ cells in control and treated cells (with vehicle-treated control cells set to 100%) for OPC cultures. The number of positive cells was counted in 20 optical fields under a 20× magnification. Data are the mean ± S.E. of three independent experiments. *p < 0.05; **p < 0.01 compared to control; non-parametric Mann Whitney test. **(F)** Histograms show the quantification of the myelin segments, calculated as the ratio between the white pixels area and the green pixels' area (Myelination Index), for OPC-DRG co-cultures. Data are the mean ± S.E. of the index obtained from the analysis of six random fields of three coverslips for each experimental condition from three independent experiments. *p < 0.05, Student's *t*-test.

Tests in the previous paragraphs have demonstrated proportionality between the affinity data evaluated, by docking chemical compounds to the binding site of a GPR17 structure shaped by homology modeling, and binding data measured *in vitro*, with such an instrumental approach as SPR in which only the target protein is involved. The further development was to demonstrate proportionality between the above findings and the biological response. The first step involved an *in vitro* test performed on cells and measuring an early effect of the downstream signaling pathway activation. The effects of Asinex 1 on adenylyl cyclase activity was evaluated in primary purified OPC cultures. In line with previous data obtained for GPR17 endogenous agonists, also the synthetic compound Asinex 1 concentration-dependently inhibited forskolin-stimulated cAMP formation with an EC50 value of 6.5 ± 0.8 nM (Figure 4A). Moreover, this effect was competitively antagonized by Cangrelor (IC50 10.2 ± 0.8 nM), suggesting a specific involvement of GPR17 (Figure 4B). These results are consistent with data reported by our group in cells transfected with GPR17 (Eberini et al., 2011) and further confirm that Asinex 1 interacts with the same GPR17 nucleotide binding site recognized by the endogenous ligands UDP and UDP-glucose (Ciana et al., 2006).

#### Effects of GPR17 Activation by Asinex 1 on OPC Maturation and Myelination

The second step was again an *in vitro* test in culture but involved cells of two different lineages and targeted a more complex and more delayed outcome of GPCR stimulation. Thus, we evaluated whether Asinex 1 can promote myelination *in vitro*, based on previous data showing that GPR17 activation by endogenous ligands accelerates OPC differentiation toward a mature phenotype (Fumagalli et al., 2011). In order to verify whether also the synthetic GPR17 ligand Asinex 1 has myelinating properties, we tested this compound in purified OPCs, cultured alone and in the presence of DRG neurons. The endogenous ligands UDP and LTD_4_ were selected as reference compounds. As shown in Figure 4, Asinex 1, tested at nanomolar concentrations, strongly increased the percentage of mature myelin basic protein (MBP^+^)-cells in primary OPCs (CTRL: 100 ± 5.53%; LTD_4_ 200 nM: 171.0 ± 21.70%; ASN1 1 nM: 166.5 ± 16.21%; ASN1 10 nM: 158.3 ± 15.77%) (Figure 4C), and promoted the formation of myelinated axons, as shown by increased “Myelination Index” in OPC-DRG co-cultures (Figure 4D) (CTRL: 100 ± 12.10%; UDP: 193.9 ± 32.31%; ASN1 10 nM: 240.3 ± 64.43%), compared to vehicle-treated control. Overall, these data indicate that, by acting on GPR17 receptor, Asinex 1 is able to foster OPC maturation, as also confirmed by appearance of a myelinating phenotype in culture (Figures 4E,F).

## Discussion

SPR is a versatile and powerful technique for measuring the direct binding between an immobilized protein and its ligands. It is emerging in the last years as one of the most popular approaches in fragment-based drug discovery, due to the possibility to perform rapid and cost-effective high throughput screenings of fragment libraries. Among others, a great advantage of this method is the need for extremely low amounts of both target proteins and ligands, which makes it very useful in case of proteins, such as membrane protein, that are difficult to produce and purify. Several strategies have been developed to immobilize membrane proteins onto the sensor chip, including trapping the protein on a hydrophobic surface mimicking a membrane-like environment (e.g., lipid bilayers or nanodiscs) (Karlsson and Löfås, 2002; Glück et al., 2011). Alternatively, the tagged receptor can be captured by a specific antibody immobilized on the chip surface or via Ni-mediated affinity capturing (Chu et al., 2015).

A major issue in SPR analyses of membrane protein is the retention of the native conformation and binding properties of the immobilized target, and this is particularly true for GPCRs. Here we have successfully immobilized two engineered variants of the GPR17 receptor designed for crystallographic studies, GPR17-T4 1–339 and GPR17-T4 16–339, and have analyzed their binding with two high affinity ligands: the antagonist Cangrelor and the agonist Asinex 1. The two GPR17 constructs can be effectively extracted and solubilized in detergent solution, and retain good monodispersity and activity even in the ligand-free form, a mandatory prerequisite for SPR analysis. We set up a protocol for single-step immobilization of the receptor on the sensor chip through a covalently-bound anti-His_6_-antibody. The protein was captured directly from the detergent solubilized membrane extracts, avoiding the costly and time-consuming purification of the receptor. The receptor retained its ligand binding activity for over 24 h when immobilized on the chip, thus allowing the design of fast screening experiments with fragment libraries (i.e., with a single injection for each compound) for the identification of new potential ligands. Furthermore, the use of a covalently immobilized antibody makes possible, after a mild regeneration step to remove the bound receptor, to reuse many times the same chip just re-capturing a new batch of freshly solubilized protein.

The affinity constants of the engineered full length receptor (GPR17-T4 1–339) that we have determined by SPR are in good agreement with that previously estimated for both ligands with an indirect binding assay ([^35^S]GTPγS) on in mammalian cells transfected with wild-type GPR17. It is worth noting that the affinity of GPR17-T4 16–339, lacking the first 15 residues, is substantially lower (about 50 times) compared to that of the full-length variant, hinting to a functional role of some N-terminal residues in ligand binding. The involvement of few residues of this region (Leu4 and Val6) in ligand recognition was already proposed in a previous computational study aimed at identifying new ligands for GPR17 (Eberini et al., 2011). Overall, our new docking/MD simulations, together with the SPR experimental results, strengthen this hypothesis and allow us to speculate on the importance of the N-terminal on GPR17 signaling. Of note, the relative ranking of the affinities of the two ligands obtained through the different assays, is inverted. This might be due to the different sensitivity of the various assays in which the affinity is measured, i.e., computational simulations in a 3D model or SPR experiments in a non-physiological system. Moreover, as already reported, the use of empirical scoring functions for estimating dissociation constant values has accuracy in the range of one order of magnitude (Eberini et al., 2008; Galli et al., 2014).

To confirm the validity of our approach for the identification of new GPR17 ligands to be exploited *in vivo* in demyelinating disorders, we performed a series of functional assays on a cell system natively expressing GPR17, e.g., primary rat OPCs grown in culture alone or in co-culture with neurons (Fumagalli et al., 2011, 2015). Our data confirm that Asinex 1 is indeed a full agonist at GPR17 acting on the nucleotide binding site of the receptor (Eberini et al., 2011), as shown by the inhibition of cAMP formation, the antagonism by Cangrelor, and the ability to promote OPC maturation and differentiation.

Taken together, the results presented in this work demonstrate that our protocol provides an effective and reliable way to measure the direct binding of GPR17 even in the sub-nanomolar range, and will be implemented for the systematic identification of new active compounds on this important pharmacological target. More generally, our findings confirm the potential of this technique, in combination with complex *in silico* analyses and functional assays on native systems, for evaluating the activity of agonist and antagonist ligands on GPRCs, a family of crucial targets in pharmacological research.

## Data Availability Statement

The datasets generated for this study are available on request to the corresponding author.

## Ethics Statement

The animal study was approved by the Animal Care Committee of the Università degli Studi di Milano–La Statale, which is legally entitled for the use of animals for scientific purposes, and by the Italian Ministry of Health (Authorization #473/2015-PR, 05/06/2015).

## Author Contributions

DC and RM performed the SPR experiments. GP designed the SPR experiments and analyzed the data. CP performed all the molecular dynamics analyses and contributed to the set-up of protocols for cell lysis. SS helped with the molecular dynamics analyses and contributed to the set-up of protocols for cell lysis. IE contributed to the molecular dynamics analyses and discussed the results. MF and EB performed the *in vitro* experiments and immunocytochemistry on OPC and OPC-neuron co-cultures and analyzed the results. SD and MT performed the cAMP assays on OPC cultures and analyzed the results. MA discussed the experimental plan and results. SCe contributed to the set-up of protocols for cell lysis, discussed *in vitro* results. MR and CT critically reviewed the work. SCa designed and performed the expression of the recombinant engineered receptor. EC provided substantial contribution to the design of the work. SCa wrote the paper with inputs from the other authors. All authors contributed in reviewing the manuscript.

### Conflict of Interest

The authors declare that the research was conducted in the absence of any commercial or financial relationships that could be construed as a potential conflict of interest.
