# Supplementary material for: Surface Plasmon Resonance as a Tool for Ligand Binding Investigation of Engineered GPR17 Receptor, a G Protein Coupled Receptor Involved in Myelination
Source: Front Chem. 2020 Jan 10;7:910. doi: 10.3389/fchem.2019.00910 (PMC6966494; doi:10.3389/fchem.2019.00910)
Supplement: Supplementary file 1 [file Data_Sheet_1.PDF]

Supplementary Material

Surface Plasmon Resonance as a tool for ligand binding investigation of engineered GPR17 receptor.

Davide Capelli[1$], Chiara Parravicini[2$], Giorgio Pochetti[1], Roberta Montanari[1], Caterina Temporini[3], Marco Rabuffetti[4], Maria Letizia Trincavelli[5], Simona Daniele[5], Marta Fumagalli[2], Simona Saporiti[2], Elisabetta Bonfanti[2], Maria Pia Abbracchio[2], Ivano Eberini[2], Stefania Ceruti[2], Enrica Calleri[3] * and Stefano Capaldi[6]*

[1] Istituto di Cristallografia, Consiglio Nazionale delle Ricerche, Via Salaria km. 29.300, 00015, Monterotondo Stazione, Rome, Italy.
[2] Department of Pharmacological and Biomolecular Sciences, Università degli Studi di Milano, Via Balzaretti 9, 20133 Milano, Italy.
[3] Department of Drug Sciences, University of Pavia, Viale Taramelli 12, 27100 Pavia, Italy.
[4] Department of Food, Environmental and Nutritional Sciences (DeFENS), Università degli Studi di Milano, Via Mangiagalli 25, 20133 Milan, Italy.
[5] Department of Pharmacy, University of Pisa, Via Bonanno 6, 56126, Pisa, Italy.
[6] Department of Biotechnology, University of Verona, Ca' Vignal 1, Strada Le Grazie 15, 37134 Verona, Italy.

[$] these authors contributed equally to this work

*** Correspondence:**
stefano.capaldi@univr.it, enrica.calleri@unipv.it

Supplementary methods

Oligodendrocyte precursor cell cultures

Primary oligodendrocyte precursor cells (OPCs) were isolated from mixed glial cultures prepared from postnatal day 2 Sprague-Dawley rat cortex by shaking cells on an orbital shaker at 200 rpm, as previously described (Fumagalli et al., 2015). OPCs were then collected and separated from microglia by incubation for 1 h in an uncoated Petri dish. Purified OPCs were seeded onto poly-D,L-ornithine-coated glass coverslips or plates (50 μg/mL) to a specific density according to the experimental protocol (see below), in Neurobasal supplemented with 2% B27, 2 mM L-glutamine, 10 ng/mL human platelet-derived growth factor BB (PGDF-BB), and 10 ng/mL human basic fibroblast growth factor (bFGF), to promote proliferation. After 2 days, cells were switched to Neurobasal medium lacking growth and containing 10 ng/mL triiodothyronine (T_3) to allow differentiation.

To prevent astrocytes and microglia contamination in OPC-DRG co-cultures, the immunopanning technique was utilized to eliminate non-oligodendroglial cells from the cell population obtained by shaking mixed glial cultures (see above) according to the protocol described in (Taveggia et al., 2008). The day before the shaking, two 10 cm Petri dishes were incubated overnight with 10 mL goat anti-mouse IgG (final concentration 0.5 μg/mL) diluted in 50 mM Tris HCl pH 9.5. The following day, dishes were rinsed 3 times with sterile PBS and incubated at room temperature (RT) for 3 h with primary antibody Ran-2 hybridoma supernatants (kind gift of Dr. Taveggia) diluted in bovine serum albumin (BSA) Medium containing MEM, 0.1% BSA and 2.5 % Hepes 1 N.

The cell suspension, obtained after shaking of mixed glial cell cultures for 3 hours, was transferred into a 50 mL tube and centrifuged at 1,200 rpm for 7 min. The supernatant was carefully discarded, and the pellet was resuspended and dissociated in 6.5 mL of NM15 medium containing MEM, 15% FBS, 2 mM L-Glutamine, 6 mg/mL Glucose, 5 μg/mL Insulin and Penicillin 100 U/ml-Streptomycin 100 μg/ml. The cell suspension was incubated for 20 min at RT with one of the Ran-2 coated-dishes, which were previously rinsed 3 times with sterile PBS. Non-attached cells were collected and re-incubated for further 20 min with a second Ran-2 coated-dish. After incubation, the cell suspension was centrifuged at 1,200 rpm for 10 min. Finally, the supernatant was discarded, and the pellet was resuspended in a small amount of medium containing MEM supplemented with 4 g/L Glucose, 10% FBS and 2 mM L-glutamine for cell count.

OPC-DRG co-cultures

Dorsal root ganglia (DRG) neuron-OPC co-cultures were prepared as described (Fumagalli et al., 2015). Briefly, DRGs from E14.5 mouse embryos were plucked off from the spinal cord, put in culture (1 DRG/coverslip) in Neurobasal medium supplemented with B27 in the presence of NGF (100 ng/mL) and cycled with 10 μM fluorodeoxyuridine to eliminate all non-neuronal cells. After 20 days, when neurites were well extended radially from DRG explants, 35,000 OPCs were added to each DRG and co-cultures kept in MEM supplemented with 4 g/L Glucose, 10% FBS and 2 mM L-glutamine. Myelination was induced the following day by the addition of recombinant chimeric TrkA-Fc (1 μg/mL) to the culture medium.

Immunocytochemistry

Both primary OPCs and OPC/DRG co-cultures treated with either the selected compounds (Asinex 1 and Cangrelor) or with the endogenous GPR17 ligands were fixed at RT with 4% paraformaldehyde (in 0.1 M PBS containing 0.12 M sucrose). Labelling was performed by incubating cells overnight at 4 ºC with the following primary antibodies in Goat Serum Dilution Buffer (GSDB; 450 mM NaCl, 20 mM sodium phosphate buffer, pH 7.4, 15% goat serum, 0.3% Triton X-100): rat anti-MBP (Millipore), rabbit anti-NG2 (Millipore), rabbit anti-GPR17 (Cayman Chemicals), mouse anti- SMI 31 and mouse anti SMI 32 (Cell Signaling). Cells were then incubated for 1 h at RT with the secondary goat anti-rat,

goat anti-rabbit, or goat anti-mouse antibody conjugated to Alexa Fluor 555 or Alexa Fluor 488 (Life Technologies). Nuclei were labelled with Hoechst 33258 (1:10,000; Life Technologies). Coverslips were finally mounted with DAKO fluorescent mounting medium and analyzed under a fluorescence or confocal microscope (LSM510 META, Zeiss). For cell count, 20 fields were acquired at 20X magnification (at least three coverslips for each experimental condition) under an inverted fluorescence microscope (200M; Zeiss) connected to a PC computer equipped with the Axiovision software (Zeiss). Images were collected and cells scored and counted using the ImageJ software.
For co-culture analysis, stacks of images of MBP and SMI31 and SMI32 positive cells were taken under confocal microscope at 40X magnification and the ZEISS LSM Image Browser was utilized to automate quantification of the myelination index. Images in the stack were merged at each level and the myelination index was calculated.

Statistical analysis
All results are expressed as mean±S.E. of at least three independent experiments. Statistical analysis was done with non-linear multipurpose curve-fitting Graph-Pad Prism program. The statistical test used was chosen according to the type of experiment performed and was indicated in the legend to figure. Three degrees of significance were considered: $P < 0.05$ (*), $P < 0.01$ (**), $P < 0.001$ (***).

Supplementary reference

Taveggia C, Thaker P, Petrylak A, Caporaso GL, Toews A, Falls DL, et al. Type III neuregulin-1 promotes oligodendrocyte myelination. Glia. 2008 56(3):284-93.

Supplementary figures

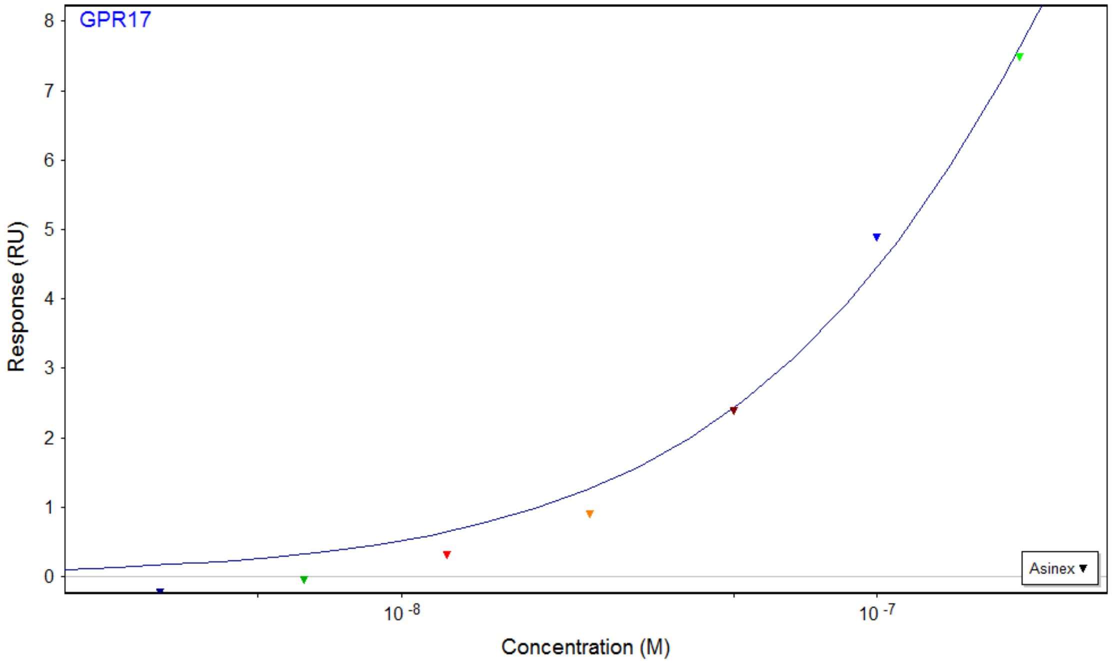

Supplementary Figure S1. Dose response plot of Asinex 1– GPR17 T4 1-339 (analyte concentrations (nM): 3.125, 6.25, 12.5, 25, 50, 100, 200).

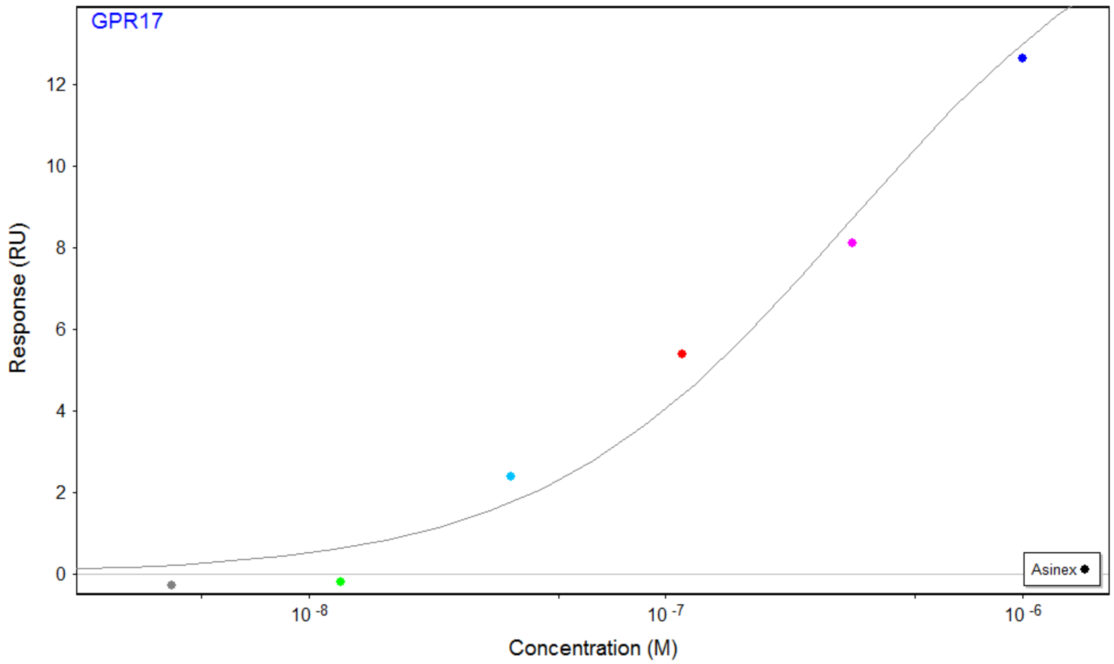

Supplementary Figure S2. Dose response plot of Asinex 1– GPR17 T4 16-339 (analyte concentrations (nM): 4.1, 12.3, 111.37, 333, 1000).

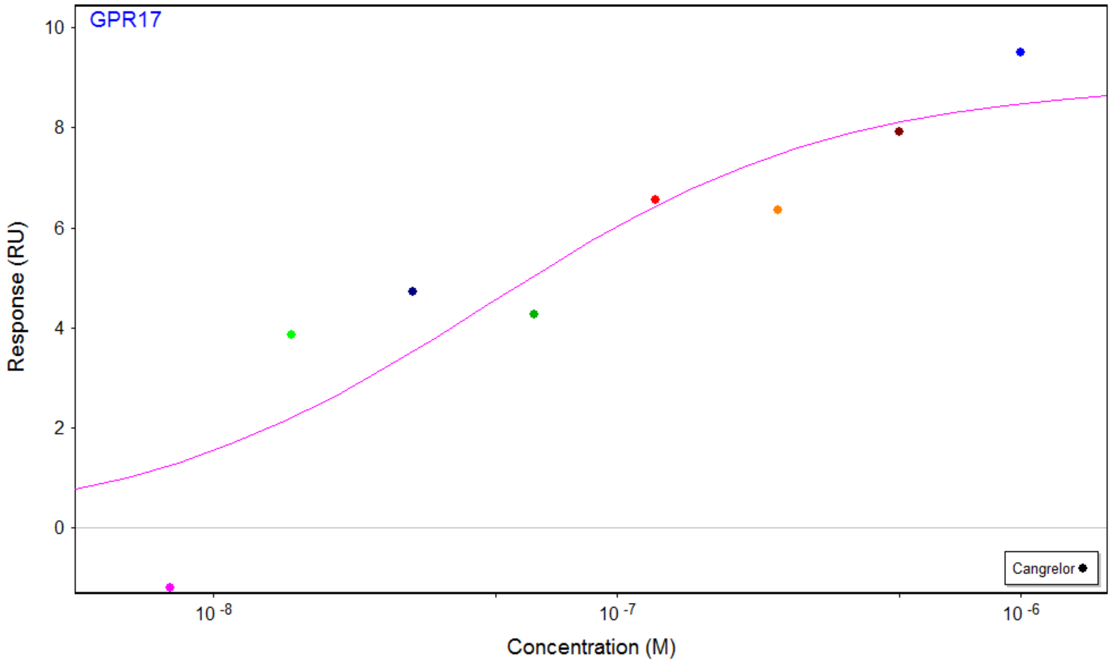

Supplementary Figure S3. Dose response plot of Cangrelor – GPR17 T4 1-339 (analyte concentrations (nM): 7.81, 15.62, 31.25, 62.6, 125, 250, 500, 1000).

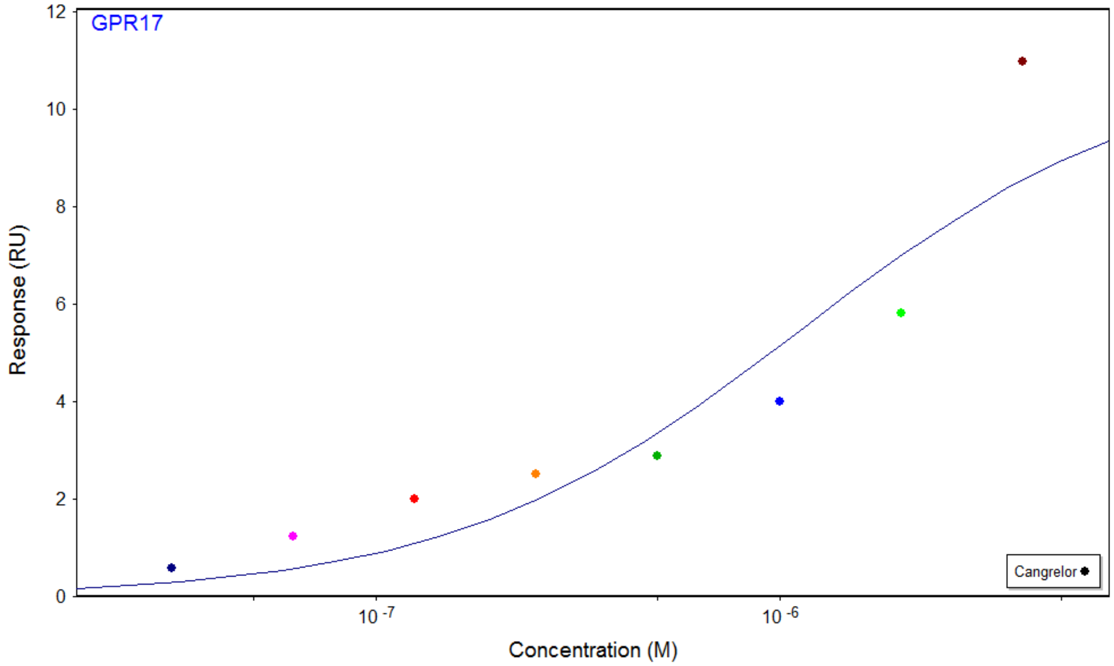

Supplementary Figure S4. Dose response plot of Cangrelor – GPR17 T4 16-339 (analyte concentrations (nM): 31.25, 62.5, 125, 250, 500, 1000, 2000).

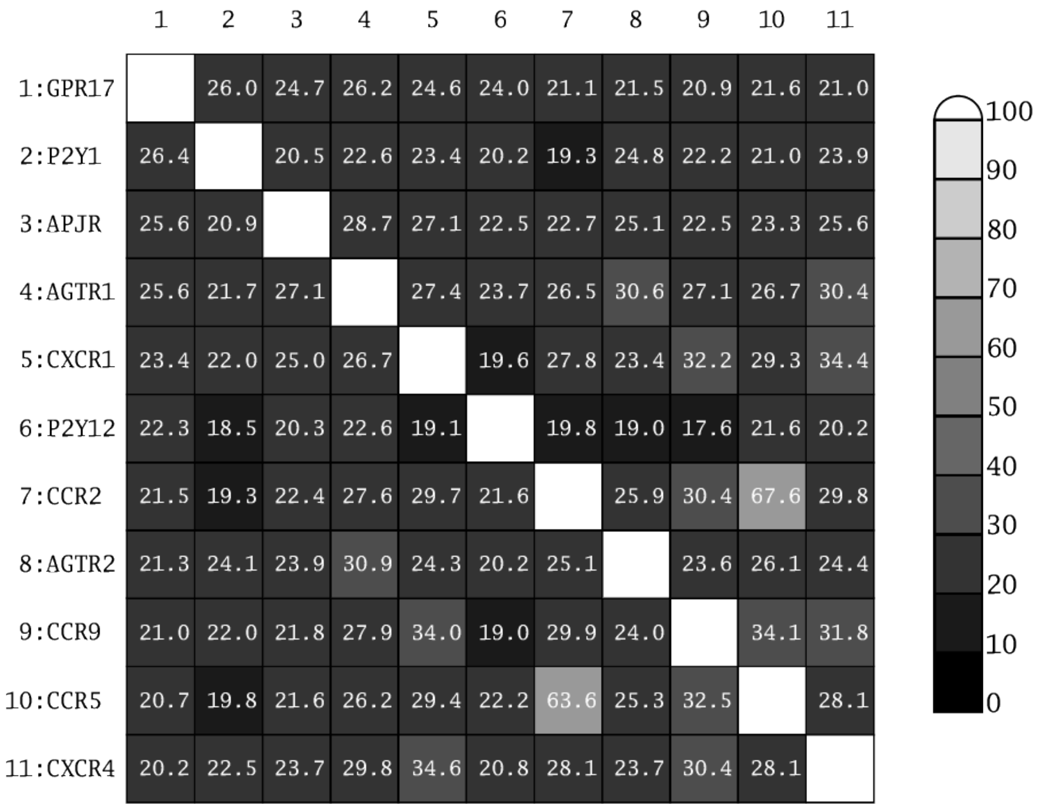

Supplementary Figure S5. Pairwise identity matrix between GPR17 and the structurally related class-A GPCRs crystallized so far. Primary structures of class-A GPCRs related to GPR17, for which experimental structures are available in the Protein Data Bank: P2Y purinergic receptor 1 (P2Y1), P2Y purinergic receptor 12 (P2Y12), C-X-C chemokine receptor type 1 (CXCR1), C-X-C chemokine receptor type 4 (CXCR4), C-C chemokine receptor type 2 (CCR2), C-C chemokine receptor type 5 (CCR5), C-C chemokine receptor type 9 (CCR9), apelin receptor (APJ), type-1 angiotensin II receptor (AGTR1), and type-2 angiotensin II receptor (AGTR2). Identity scores are computed for the selected GPCRs according to a previously published alignment (Parravicini et al., 2016; Sensi et al., 2014) and sorted based on their decreasing sequence identity against GPR17. The table value at row i, column j equals the number of residues, matches between sequences i and j, divided by the length of sequence j.

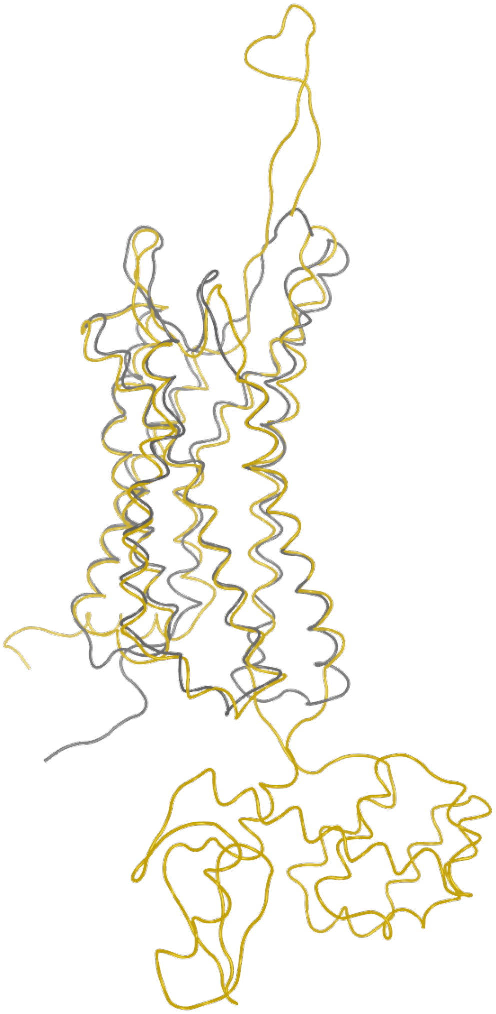

Supplementary Figure S6. Superposition between GPR17 homology models. The homology models computed for GPR17 receptor and its engineered variant GPR17-T4 1-339 are represented as gray and yellow tubes, respectively. Only the Cαs of the TM bundle were used as reference coordinates for structural alignment.

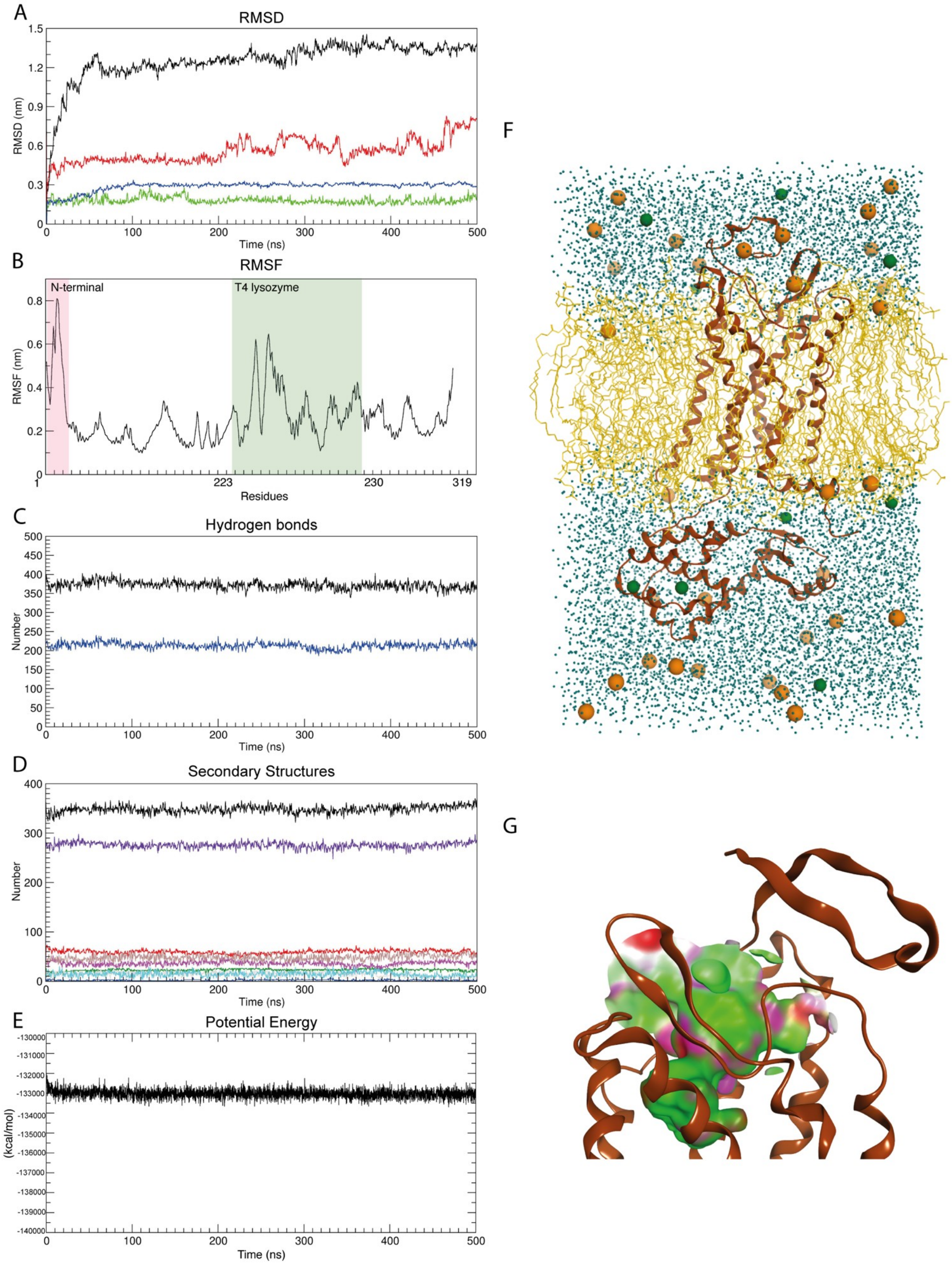

Supplementary Figure S7. Evolution of geometric and thermodynamic parameters of the GPR17 closed conformations over molecular dynamics (MD) time. A) Root Mean Square Deviation computed for Cα carbons for the whole GPR17-T4 1-339 protein (black), for the helical bundle (blue), for the T4 lysozyme (green) and for N-terminal region (red). B) Root Mean Square Fluctuations computed for Cα carbons. C) Number of hydrogen bonds for the whole protein (black) and for the helices (blue). D) Number of secondary structures: total number (black), coil (red) β-sheet (green), bet-bridge (blue), bend (magenta), turn (brown), α-helix (purple), 5-helix (grey), 3-helix (cyan). E) Potential energy. F) Whole MD system, including the engineered variant GPR17-T4 1-339 (brown ribbons), POPC (yellow sticks), water (petrol sticks), Cl^- ions (green spheres), Na^+ (orange spheres). G) Van deer Waals molecular surface computed for the binding pocket of a representative GPR17-T4 1-339 closed conformation, colored according to the presence of polar (purple), hydrophobic (green) and solvent exposed residues (red).

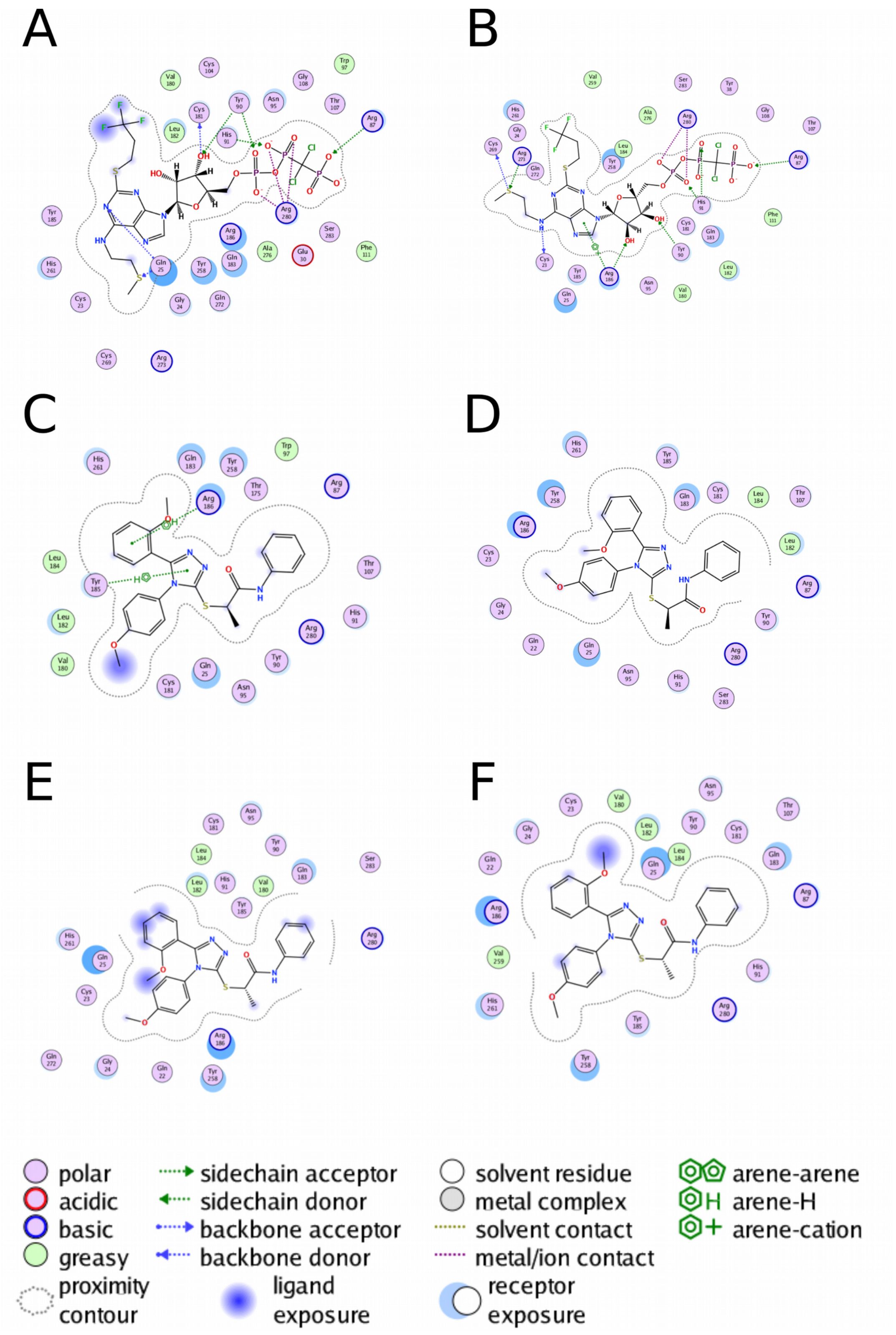

Supplementary Figure S8. 2D interaction plots. Ligand::receptor interactions computed for the top-scoring docking complexes obtained for Cangrelor (A, B), Asinex 1 (R enantiomer) (C, D) and Asinex 1 (S enantiomer) (E, F), in the open (A, C, E) and closed (B, D, F) N-terminal conformation.
